# Online collaborative Padlet-mediated learning in health management studies

**DOI:** 10.3389/fpsyg.2023.1157621

**Published:** 2023-03-30

**Authors:** Lior Naamati-Schneider, Dorit Alt

**Affiliations:** ^1^Health Systems Management Department, Hadassah Academic College, Jerusalem, Israel; ^2^Faculty of Education and Instruction, Tel Hai College, Upper Galilee, Israel

**Keywords:** online collaborative learning, Padlet, health management, higher education, collaborative learning

## Abstract

**Introduction:**

The Padlet interactive platform constitutes a virtual online board on which users can post various types of multimedia content, such as documents, questions, comments, images, video clips, and audio clips. This platform has gained popularity in higher education yet remains ancillary in the fields of medical education and medical management.

**Methods:**

This case study sought to initially design an intervention program that employs online collaborative learning enabled by Padlet and to assess how Health Management students perceived the activity and its ramifications for their learning. 85 students’ reflective journals were content analyzed.

**Results:**

five main categories stood out: (1) user experience of the platform; (2) visuality and visibility of the platform; (3) collaborative learning and OCL; (4) active online learning and student engagement; and (5) cognitive flexibility.

**Discussion:**

This study emphasizes the importance of incorporating digital tools in education, particularly the use of the Padlet platform to facilitate collaborative learning and improve the quality of teaching and learning. It demonstrates that Padlet-mediated online collaborative learning can be an effective digital learning tool because of its ease of use and ability to accommodate both pedagogical and technological challenges.

## Introduction

The healthcare industry has faced numerous challenges in recent years due to rapid global changes, increased access to information, and evolving labor market demands. As a result, the training of healthcare administrators must be competency-based and focus on developing essential skills such as cognitive and interpersonal abilities (Senkubuge et al., 2014; Naamati-Schneider, 2020). The dynamic nature of healthcare systems, which involves interactions between individuals and their environments, requires teaching methods that can help healthcare providers adapt to the changing demands of the industry (Reis, 2019).

This study proposes the evaluation of the effectiveness of online collaborative learning (OCL) as a teaching method for health management students, utilizing Padlet as a digital platform for interactive collaboration. Previous research in the field of health professions, including health administration, has demonstrated that OCL in digital environments can enhance student learning through collaborative problem-solving and the acquisition of knowledge, as well as the cultivation of higher-order thinking skills (Xiong et al., 2016).

The acquisition of skills such as flexibility, effective decision-making, and interpersonal communication is crucial for health professionals, who often work in multidisciplinary and culturally diverse teams. These skills can be developed through exposure to online collaborative learning (OCL) environments early on in professional training (Männistö et al., 2020). The adoption and advancement of OCL pedagogies across all levels of education, from undergraduate studies to professional development, is widely believed to benefit both the health system and its patients by enabling health workers to meet the demands of the healthcare market (Alt et al., 2022). This is particularly relevant in light of the practical nature of work in the health industry (Grosser et al., 2020).

The current study hence sought to demonstrate the introduction of an innovative pedagogy of online collaborative learning (OCL) with the aid of Padlet technology (Ramachandiran and Mahmud, 2018), for health management undergraduate students. Our aim was to examine students’ perceptions of their experiences of OCL enabled by Padlet from their point of view. Indeed, students’ perception of the use of technologies and digital platforms has been found to be an important factor in the extent of the adoption of technology and the achievement of the learning goals (Mehta et al., 2021), therefore scholars have been called upon to extend the knowledge in this field.

In this study, we analyzed students’ reflective journals with the aim of understanding how the students perceived the activity and its ramifications. Content analysis helped identify themes and patterns regarding students’ perceptions of the newly designed activity. The findings of this study will help in understanding the benefits of Padlet as a collaborative learning tool, and how it should be implemented so as to develop the ability to work in varied teams, to collaborate in decision-making, and to adapt to changes in the learning environment. With these skills and abilities, students will undoubtedly be better able to adapt to the labor market in health professions and in general (Männistö et al., 2020; Alt et al., 2022).

## Literature review

In this section we will review the advantages as well as challenges of OCL. We will also address Padlet-mediated learning in technology-enhanced environments by reviewing relevant recent literature. Further, we will present the use of this teaching methodology in the healthcare professions and examine how it impacts the skills required of today’s healthcare students.

### Online collaborative learning

Zheng et al. (2014) defined online collaborative learning (OCL) as a goal-oriented activity of a group of students committed to achieving a shared goal and creating new knowledge by learning interactively in a digital environment. This methodology has become widespread because of its many advantages. For example, studies show that encouragement of students’ engagement, high accessibility to the course material (Sukendro et al., 2020; Balouchi and Samad, 2021), extensive access to colleagues and instructors, and encouragement of interaction and collaboration among the students contribute to better learning outcomes (Ornellas and Muñoz Carril, 2014). OCL was found to add interest and to raise motivation to conduct research and study, and as such to encourage the transition from passive to active learning, and to encourage interaction and connection between the students through the sharing of the products of their study and new ideas, leading to greater social engagement and contributing to the development of higher-order intellectual skills (Malik et al., 2017; Park et al., 2019; Yadegaridehkordi et al., 2019).

Various studies (Ng et al., 2022) have explored the factors that affect the degree of students’ adoption of OCL. For example, Cole et al. (2021) showed that students’ perceptions of the online learning environment were an important predictive factor in their engagement in learning by means of online courses. Studies have also found that the nature of the social interaction in the learning environment, support and positive interaction with the facilitator, and the type of technology used are considered key factors in the degree of adoption of OCL and the extent to which it influenced the achievement of the learning goals and improvement of performance (Molinillo et al., 2018; Qureshi et al., 2021). Other studies evaluated the outcomes of OCL and examined their level and quality. Breen (2015) and DiPasquale (2017), for example, presented means of evaluating the outcomes of OCL by analyzing the initial stages of the establishment of the higher-order learning outcomes by means of brainstorming, group discussion, and presenting various ideas, orally or in writing. Blieck et al. (2019) pointed to the development and encouragement of ideas as outcomes of higher-order learning with this methodology and the guidance and support of the instructor. Similarly, Aldholay et al. (2018) demonstrated the development of higher-order thinking by means of processing, reflection, and analysis of collaborative critical thinking regarding specific topics relevant to the course content. This supportive and collaborative learning environment enables the existence of an effective learning perspective for attaining the maximum learning goals.

### Online collaborative learning mediated by Padlet

The Padlet interactive platform constitutes a virtual online board on which users can post various types of multimedia content, such as documents, questions, comments, images, video clips, and audio clips. The platform and the board are available to learners throughout their studies and even beyond (Rosnida and Zainor, 2018; Mehta et al., 2021). Recent studies have shown the contribution of Padlet-mediated learning and its usefulness in encouraging learners by means of active teaching and collaborative learning (Rosnida and Zainor, 2018; Rashid et al., 2019).

According to the study of Gasmi and Thomas (2017), this technology has been seen to promote collaboration among learners and to promote students’ engagement in seminar courses. The study found that the technology contributed to the efficiency of their studies and constituted an easy and secure learning environment for communication, teamwork, and asking questions. These studies shed a positive light on the use of Padlet-mediated collaborative learning. They note the advantages of the tool in its ease of use, accessibility to materials over time (even after the conclusion of the course), and work with diverse resources (including links, images, and video clips), which make it a convenient tool for student-focused active collaborative learning (Mehta et al., 2021).

Several studies have demonstrated the benefits of using Padlet as a tool for collaborative learning. Garnham and Betts (2018) found that student engagement increased in seminar courses that utilized Padlet, and Beltrán-Martín (2019) reported that students experienced satisfaction and improved academic performance when using the platform. Padlet has been identified as having several advantages for collaborative learning, including ease of use, visual simplicity and long-term accessibility of content, even after the end of the course. These advantages of the Padlet created a friendly tool for users that led to high student satisfaction and better performance. Moreover, Padlet is a very easy-to-operate technological tool that students can use with various apps and resources such as presentations, links, images, and videos (Beltrán-Martín, 2019).

In the contemporary academic landscape, the availability of various technological tools has become an essential aspect for students to present their knowledge and communicate their ideas. However, while these multiple options provide numerous opportunities, they may also pose a challenge, as learners must devote significant time and effort to acquire the necessary technological skills. As a result, the Padlet can prove to be advantageous in this regard. Furthermore, these features allow for a holistic and student-centered approach to teaching and learning, as students are able to use their preferred medium and become actively involved in creating their own content (Mehta et al., 2021).

The literature review indicates that Padlet-mediated OCL methodologies are not yet widely used in higher education in the fields of medical education and medical management and that there is little research on how the methodologies are used in higher education. Therefore, the current study aimed to implement an intervention program (described below), that combined Padlet-mediated OCL. Next, it sought to evaluate the perception among health management students of its effectiveness in collaborative learning. Mapping the students’ perceptions and understanding the effects of using this technology-mediated methodology is essential for achieving learning goals and acquiring the requisite skills, especially in studies that examine the students’ perspectives, because of the difficulty in tracing students’ engagement in online learning activities (as opposed to face-to-face learning encounters). The data from such studies can be important for evaluating the effectiveness of the learning methodology (Mehta et al., 2021). The current qualitative study is based on a thematic analysis of health management students’ reflective journals that were submitted at the end of the learning process. It presents the challenges and opportunities of the proposed learning method as viewed by the students.

## Method

The current study uses the case study method. In this method, as defined by Yin (2014), there is an in-depth empirical investigation of the current phenomenon and its relation to the real world. Data are gathered regarding human activities in a specific time and place, enabling the learning of several things about the processes that take place in the case under research. In a qualitative study in general and in case study research in particular, the researcher constructs themes that arise from the participants’ words in such a manner that an argument or generalization derived inductively from the participants’ words receives empirical support from quotations (Cresswell, 2009).

### Participants

Data for the analysis were gathered from 85 Israeli undergraduate students of a Health Management program (covering patient-doctor relations, quality of service in the healthcare system, and ethics and patient rights). The students’ mean age was *M* = 25.70 (*SD* = 5.85), and 84% were females. In relation to ethnicity, 58% were Jews, and 42% Arabs (Muslims). Thirty-one percent reported working in health professions (*Profession*).

### Data gathering

The students were asked to keep a reflective journal throughout the course and to submit it at the end of the semester. They were instructed to keep a record of the learning process as they saw it and to describe the challenges, difficulties, and positive outcomes in light of their experience (Tripto et al., 2016). For example, they were asked to describe the main challenges, the difficulties with which they had coped, and the positive and negative experiences of the process. Keeping the journals enabled reflection regarding the significant learning processes and adoption of a multidimensional view of this process (Zohar and Barzilai, 2013).

Data were collected through the reflective diaries, following the intervention plan described in the next section (see the Instructional Method). Prior to its implementation, and for the purpose of maintaining the ethical principles concerning research with humans, the following topics were kept among others: The theme of the research was clarified to the students. It was clarified that the reflective diaries will be used to evaluate the intervention plan. The students’ consent was obtained by signing an “informed consent” form. The participants were assured that every name would be replaced by a pseudonym and every identifying item would be omitted when writing the research report. It was made clear to the students that the materials (the reflective diaries) would be processed anonymously only. The research was approved by the college’s Ethics Committee.

### Procedure of the instructional method (intervention plan)

Underlying this study is the transformation of a required course titled Introducing Quality into the Health System, which had been taught in a traditional frontal manner, to a course using a methodology of Padlet-mediated OCL. This methodology enabled OCL that included work in small groups; building a collaborative data pool; final projects focusing on dilemmas and analyses of them in organizations, using the data pool the students had constructed; presentation of the projects on the collaborative platforms; and peer review (Levenberg and Barak, 2015; Ng et al., 2022). At the beginning of the course, the students were exposed to the new methods of teaching and received information about the Padlet technology and how to use it. The technology was also demonstrated by being used for students’ introduction. This introduction included students’ presentations of themselves using the Padlet board, thus enabling familiarity with the tool, constituting an informal and equal experience of the tool, and generating interaction between the students to create groups and increase students’ engagement in the course (DeWitt et al., 2015; Rashid et al., 2019; Qureshi et al., 2021).

The course consisted of several stages, each one based on the preceding one. After familiarizing themselves with the course outline and the technology, the students were asked to form online study groups with which they would work throughout the semester. In the second stage, the students were asked to choose a topic from among the course topics presented to them on a Padlet board. In the next stage, the study groups were asked to formulate a question, problem, or dilemma related to the selected topic and to gather information from articles on the topic. The groups were asked to present the articles and analyze them in the context of the selected topic and to post the information on a new Padlet board. This board was organized by topics and subtopics, in accordance with the selected topics. Topics selected included dilemmas in introducing accreditation processes, challenges in maintaining medical confidentiality, clinician-patient relationships through digital tools, and measuring the quality of service in the organization.

By the end of this stage, a categorical board containing information, articles, and links to academic content was conducted. This collaborative information bank was congruent with the course topics and divided in a categorical manner that was easy to navigate and accessible to all the students in all the groups (Mehta et al., 2021). In the next stage, the groups were asked to prepare and present a final project that was posted on yet another Padlet board. In this project, which was based on the preceding work, they were required to broaden the scope of the original project into a comprehensive research question or discussion of a specific dilemma in a specific organization so as to include a number of perspectives, and based on, among other sources, the collaborative knowledge pool of the entire class (and even to extend their project by adding new materials).

**Figure 1:**
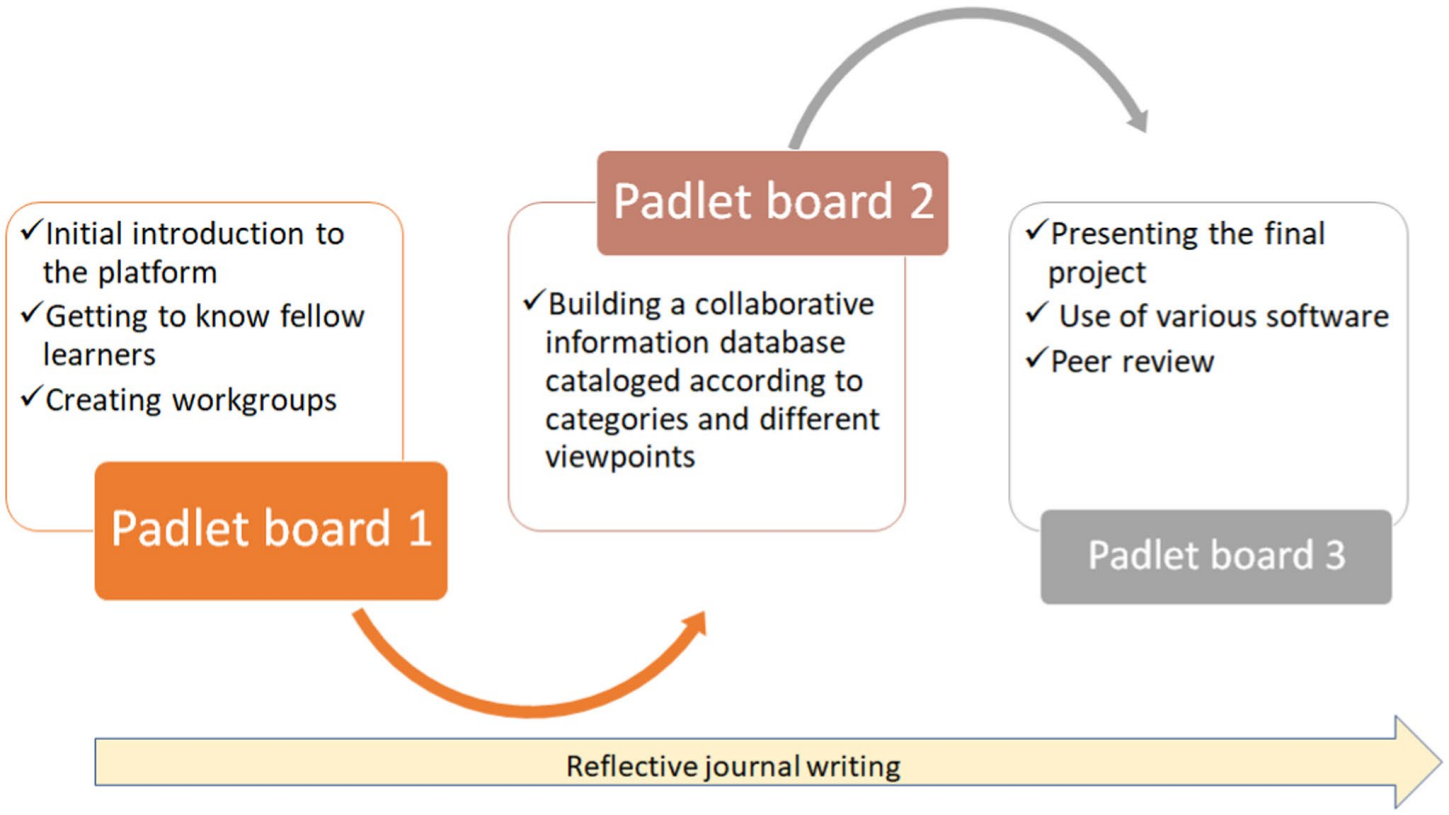
The procedure of the instructional method.

As part of the final project, the students were required to present organizational and managerial perspectives as well as the perspectives of patients and health professionals in the system. This was to be accomplished by means of online group meetings and collaborative work, including brainstorming, discussion, and decision-making regarding the construction of the final project and its visual presentation on a Padlet board, in various formats and using various technologies that they chose. These included, for example, e-posters, presentations, and video clips using such technologies as PowerPoint and ThingLink, with which they had become familiar in other courses. This enabled variety and empowerment of the individual student by allowing freedom of choice of the manner of presentation, which is an advantage of the Padlet technology (Ramachandiran and Mahmud, 2018). The third board brought together all the projects in the course. The students were required to use the Padlet board to critique their classmates’ projects exhibited on that board, ask questions, add information, or highlight topics to be added to the discussion of the dilemma. Figure 1 summarizes the procedure of the instructional method.

### Data analysis

This study used a categorical analysis of the data in accordance with the deductive- inductive approach. This combination of approaches throughout the analysis is considered essential because it enables the addressing of categories derived from the literature review and the existing theoretical knowledge in the field combined with new categories that arise from the data analysis (Cresswell, 2009). Thematic analysis was used in this study to identify topics that appeared in the data and to report them. Each theme was summarized to provide an overview of the participants’ reports. Then the dominant topics in the reflective journals were identified and were gathered together as main categories (Braun and Clarke, 2006). Disagreements and differences between the researchers were resolved through discussion. The themes regarding which the disagreement was not resolved were presented to a researcher not connected with the study who determined whether the theme was established in the data. Episodes, thoughts, and feelings that the students raised are reported here in order to support the reliability of the themes found. The external validity was derived from the congruence of the themes with the knowledge found in the theoretical literature, as we show in the discussion.

### Findings

**Figure 2:**
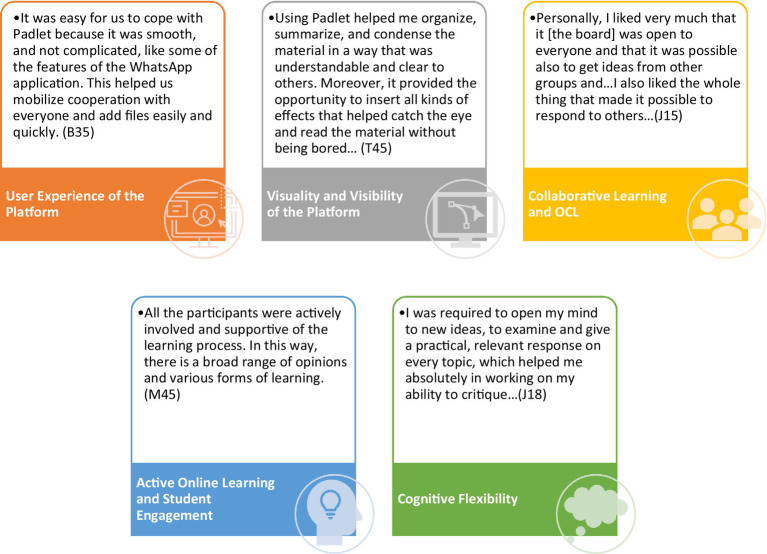
Derived categories and exemplary quotes from the data.

The data analysis of the reflective journals revealed five main categories: (1) user experience of the platform; (2) visuality and visibility of the platform; (3) collaborative learning and OCL; (4) active online learning and student engagement; and (5) cognitive flexibility. These categories are summarized in Figure 2. Exemplary quotes from the data are provided to illustrate each category.

### User experience of the platform

The analysis of the students’ journals revealed that the use of this interface was easy for the students and enabled a positive experience. Most of the students noted the platform’s ease of use and the fact that it did not require much learning and experience; after a relatively brief explanation, it was possible to use it freely. In this way, the platform makes the user experience easy and pleasant.

It was very easy in my opinion, and therefore also made it [the Padlet assignments] very enjoyable. They were almost easier and more enjoyable than all the other assignments in other courses. (B22)

Many of the students noted that for them, the platform was almost as experiential as using social media and familiar, favorite applications that they use for online social interaction. This experience contributed to confidence in using the tool that was familiar and informal, reducing resistance and anxiety in carrying out the assignments.

It was easy for us to cope with Padlet because it was smooth, and not complicated, like some of the features of the WhatsApp application. This helped us mobilize cooperation with everyone and add files easily and quickly. (B35)

The students’ positive user experience was addressed also in terms of the platform’s availability, which was an important element in this category. The fact that the learning and the tools were digital enabled high accessibility to all the material at all times and in every place, including the use of students’ phones.

The Padlet was very helpful, I quickly found an article that was relevant to my project and while I was searching, I already understood what I was going to say. The information was accessible every hour of the day. (B11)

### Visuality and visibility of the platform

Many students noted positively the visuality of the platform and the fact that it can be changed according to the students’ and instructors’ preferences. For example, the background of the Padlet and its organization could be determined by the instructor, but each group may choose the format whereby materials are added.

Using Padlet helped me organize, summarize, and condense the material in a way that was understandable and clear to others. Moreover, it provided the opportunity to insert all kinds of effects that helped catch the eye and read the material without being bored. Colors and the appearance of every post helped me remember and connect [the topic] to an article that needed to be used for the second assignment. In addition, using Padlet helped me very much in choosing the material that was most important for others to read, instead of putting long and boring material. Personally, when I read the posts I was attracted to what drew my eye. (T45)

Nevertheless, a few students noted that the colorfulness and visuality of this tool were perceived as creating visual overload and difficulty in organizing the material.

In my view, the main difficulty was manifested in the visibility of the Padlet, On the one hand it is very friendly and that is colorful and divided into various windows. Here the problem begins, when I felt an overload of information at a certain stage, and it was no longer pleasant to look at and primarily made it difficult to follow the projects of other people. (C12)

As the semester and the students’ use of the platform progressed, students reported that they had honed these abilities and that they were able to cope with the ensuing assignments with fewer difficulties in this respect. They noted that these abilities continued to improve with the use of the platform and with comparisons and receipt of help and comments from group members or from members of other groups.

By the final project, we already understood better what is of secondary importance and what is of primary importance and how exactly to condense [it] so that it will catch the eyes of our classmates so that they will want to read our project. (D5)

### Collaborative learning and OCL

The experience of collaboration contributed to both the learning level and the social level. Thus, at the learning level, for example, the construction of a collaborative pool of papers and the division of the material and its exposure to all the students made it easier for them in the processes of searching for and locating material and even sorting it.

The pool of articles on Padlet is excellent, broad and truly useful and helpful. Usually when I am looking for an article for other academic projects, just finding the article is a project in itself. Thanks to Padlet, the search is fast and easy. The user experience with Padlet was enjoyable and quick and I even found several articles from which to choose. (F67)

On the social level, the students wrote in their journals that this learning methodology enabled learning in groups and comparison, critiques by peers, and learning from classmates in a positive and less threatening way.

Personally, I liked very much that it [the board] was open to everyone and that it was possible also to get ideas from other groups and … I also liked the whole thing that made it possible to respond to others and to add other things that could enrich the knowledge of the topic of my [project] or to connect to it some kind of creativity, whether a video clip or a survey or a picture. (J15)

In their reflective journals many students noted their initial concern over posting material that included assignments. Some reported fearing criticism. Those who had low self-confidence, were concerned about being criticized because of language difficulties (in a multicultural class room), or were concerned over quality.

There is, for example, concern that a project that is posted on the Padlet is not good enough, and then a feeling of doubt about the work is created and thoughts that “maybe yours is less good than what the others did” begin. (I61)

The students reported that despite their concerns about collaboration, very quickly they were exposed to a variety of projects. The possibility of revising their projects in light of the comparison with other projects or criticism from their classmates had a positive influence on their learning and on the learning product, while relating to other new ideas and other perspectives.

Sharing the projects and assignments enabled the students to critique their own projects and final assignments, compare them, and observe critiques of other projects. This enabled them to revise their own projects and thus they were exposed to a variety of ideas and perspectives as well as the implementation of a variety of abilities, such as self-criticism, and comparison and synthesis of materials.

The Padlet helped me get additional opinions of the project I submitted which would not have happened when I sent the project directly to the lecturer on the website and only the lecturer could see it. This made me understand other additional opinions of other students and to know what things to improve. (Q34)

### Active online learning and student engagement

Another category that arose from the analysis of the students’ reflective journals concerns active learning and active engagement of students throughout the course, using the Padlet digital platform. This categorical analysis revealed that the students found themselves actively involved in the learning processes and even experienced the active engagement of other students.

I feel that I had a very active learning [experience] and that it was not oppressive, which is a very big bonus. (V11)

All the participants were actively involved and supportive of the learning process. In this way, there is a broad range of opinions and various forms of learning. (M45)

In addition, there was brainstorming. That is, all the students participate in the discussion and express opinions and suggestions on a specific topic. (M11)

Students’ engagement increased the feeling of commitment to the process and the learning experience, “All the members of the group had a feeling of personal responsibility for the success of their comrades in the group and understand that their personal success depends on the group’s success.” (C33)

The students also noted that the option of adding comments and communicating about the material enabled brainstorming and discussion in writing, often over time, and led to an open discussion, editing of the material, construction of arguments, and grounding of the arguments in the material studied, individually and even anonymously, “Mostly I would [post] arguments that were based on academic articles. That opened the option of discussing with others, I had such fun using the Padlet” (F31).

### Cognitive flexibility

Another category that derives from the proposed theory concerns cognitive flexibility and its development in students throughout the intervention. The students reported being aware of interaction and solution alternatives, considered different opinions while interacting with their classmates, and were open to embrace new perspectives targeted at achieving better results. The students reported that collaborative learning enabled them to experience learning processes that included brainstorming, group discussion, ironing out of disagreements, and exchanges of ideas.

Sharing the information makes it possible to respond to it, to conduct a multi-participant discussion about certain issues in the field of health, to think about new and creative solutions. (K52)

I was required to open my mind to new ideas, to examine and give a practical, relevant response on every topic, which helped me absolutely in working on my ability to critique. Also, this tool exposed me to topics and articles I had not heard about in the past and demonstrated material for me in a broader way by providing a demonstration of the topic as it pertains to medical organizations. All this enabled me to examine information and ways of presentation from perspectives that were different from mine. In addition, during the first assignment we sat as a group and wrote responses together, which enabled us to see together and separately the different ways of thinking. (J18)

## Discussion

This study presented the process of introducing and using the methodology of Padlet-mediated OCL with students of health management. It demonstrated how it is possible to guide the OCL process using the Padlet digital internet platform, encourage collaborative learning, and develop cognitive flexibility, through the active engagement of students even in large courses, while achieving the learning goals and outcomes and higher-order thinking among the students.

The analysis of student reflective journals showed that the students found the platform to be user friendly. The term “user experience,” which in the past applied mainly to the user interface, that is, the interface between the technology and the individual, has been broadened and now refers to additional aspects such as design, presentation of information, technology, and even emotional, personal, and experiential aspects (Minichiello et al., 2018). In this context, the students noted that their user experience of the platform was positive, which encouraged them to use it. The platform even increased the interactions among the students, both because of its availability and accessibility and because of the user experience, which included attractive visuality and ease of operation and thus resembled the user experience of familiar platforms that serve in social interactions.

According to the students, the use of the Padlet platform facilitated the establishment of social and learning interactions and motivated them to maintain these relationships. The use of Padlet also promoted a positive and open atmosphere for collaborative learning, allowing for exposure to diverse viewpoints and the cultivation of flexible thinking in collaborative decision-making. This supports the findings of Cole et al. (2021), who showed that students’ perceptions of an online learning environment were an important predictive factor for student engagement in learning in online courses. In our study, the participants reported that there was a high level of activity on the part of all the students in the group, including engagement of students from various groups. The assignments were structured in a way that allowed for the accumulation of shared collaborative knowledge, which the students could then draw upon in subsequent assignments. Additionally, the platform provided a positive user experience that contributed to the success of this approach.

Together the students created a sense of collectivity and a shared goal, a desire to share knowledge, and construction of a basis for teamwork and collaboration with the other members of the small group or the entire class. The results of the present study align with previous research suggesting that the nature of interactions with the instructor and peers, as well as the user experience of the technology, are crucial factors influencing the adoption and effectiveness of OCL in achieving learning goals and improving performance (Molinillo et al., 2018; Qureshi et al., 2021). Studies of the Padlet technology specifically, such as Gasmi and Thomas’s (2017), found that the platform was perceived as promoting collaboration among the students and student engagement, asking of questions, discussions, and teamwork.

Students, who were accustomed to individual learning, initially struggled with the transition to collaborative approaches. They reported being anxious about sharing their work due to fear of criticism and lack of confidence in their abilities. However, they eventually recognized that this collaboration could be an opportunity for growth and development. With this realization, the students were able to freely provide feedback on their peers’ projects and receive comments on their own work, resulting in a positive user experience akin to that of social media rather than a threatening learning environment. These findings align with previous research indicating that OCL can promote higher-order outcomes such as brainstorming, group discussion, idea generation, organization of ideas, and critical thinking through active student participation (Breen, 2015; DiPasquale, 2017; Aldholay et al., 2018).

One particularly notable benefit of using Padlet in large classes is the ability to divide students into groups and facilitate high levels of engagement. This is in contrast to traditional compulsory courses with similar class sizes, which may struggle to allocate sufficient time for group presentations and individualized instruction for the instructor due to the size of the class (Young and Nichols, 2017). According to student journals and other research on the platform, Padlet enables highly engaged participation (Mehta et al., 2021). Therefore, the combination of this teaching method with the Padlet platform offers a solution to the challenge of effectively instructing large classes in the 21st century. This advantage is particularly relevant in the current context of social distancing measures and the shift towards hybrid learning. The use of Padlet maintains these benefits and also allows for greater accessibility in terms of both time and location, as the boards can be accessed from any internet-enabled device, including smartphones, at any time. This feature is particularly useful in the new era of education.

According to the analysis of student journals, the online methodology and the features of the Padlet platform facilitated the development of cognitive flexibility. The students reported being exposed to a variety of opinions and perspectives from their group members and the entire class, which required them to engage in written discussions, consider alternative ideas, and potentially adjust their project based on feedback from their peers. The processes described by the students align with the concept of cognitive flexibility, which involves the ability to respond adaptively and dynamically to changing circumstances, interactions, and personal experiences in order to achieve a desirable outcome (Hayes et al., 2006). It also involves the consideration of multiple approaches to a problem, negotiation, and the ability to arrive at implementable solutions in complex, varied, and multicultural environments (Boot et al., 2017). This development of cognitive flexibility is also linked to open-mindedness, defined as the willingness to consider alternative possibilities, opinions, and solutions (Barak and Levenberg, 2016).

Indeed, the students reported initial difficulty in this context, which they expressed, *inter alia*, as fear of negative criticism and low valuation, but as the semester progressed and they experienced the advantages of the methodology, this difficulty was overcome and the students began to examine new opinions and ideas, critically and objectively, and even examined their own work by comparing projects, editing, and improving while relating to the critique of their projects by their comrades. In this way they often improved the learning product and achieved higher-order learning outcomes while developing cognitive flexibility.

### Limitations of the study and future directions of research

This study utilized online collaborative learning (OCL) and the Padlet platform as tools to support a specific teaching method. While the researchers focused on the pedagogical aspect, future research should compare this digitally supported activity to a non-digital equivalent and measure the impact on students’ cognitive skills. This comparison would allow for an assessment of the specific contribution of the digital instrument to skill development and the identification of any challenges encountered. It would also be beneficial to examine the use of this instrument in different teaching methodologies to determine if it is more effective for certain types of assignments or requirements, such as the creation of concept maps or presentations of projects.

This study focused on the field of health management, but the development of these skills at the beginning of professional training in the medical field could benefit health professionals in meeting the demands of contemporary healthcare systems. As we enter a new era that requires adaptability and flexibility, it is suggested that this pedagogy be implemented in other academic fields to evaluate its impact on the acquisition of relevant skills. The results of this study highlight the need for further research on the use of digital learning environments based on learning outcomes-oriented pedagogy. This may contribute to the development of teaching strategies that foster lifelong learning skills.

## Conclusions and recommendations

The dynamic worldwide changes in the digital era and the new needs they are creating have challenged many traditional teaching methods that were widespread in academia in general and in the area of medical education in particular. Digital processes in teaching that were accelerated by the limitations of social distancing during the COVID-19 pandemic started becoming established as essential in these processes, especially because online learning processes mediated by digital platforms will leave their mark in creating the new normal.

These changes have become an opportunity for introducing and establishing innovative and active teaching methods that encourage great engagement of the learner in the learning process, instead of the traditional, passive methods that are widespread in academia in general and the fields of medicine and health management in particular. Consequently, there is a real need today for research and implementation of innovative teaching methods that are suited to the learning goals of the 21^st^ century. Developing these abilities is crucial, especially in the health professions, which are undergoing a host of changes that require the development of these skills at an early stage of training and throughout the more advanced stages, including professional training. Researchers, academics, and educators in higher education are needed to research and actively implement online learning technologies (Mehta et al., 2021).

The findings of the current study support previous studies of the use of Padlet-mediated online learning that were found to be effective in developing engagement and higher-order learning outcomes (Breen, 2015; DiPasquale, 2017; Aldholay et al., 2018) and they demonstrate specifically the effect of implementing Padlet-mediated OCL on the development of learning abilities among students of health management. The findings of this study support assumptions that combining OCL and teaching methods with digital platforms that encourage collaborative learning enable the development and acquisition of cognitive flexibility, teamwork, and higher-order learning outcomes of the kind that enhance crucial abilities of employees in health systems in the 21^st^ century (Männistö et al., 2020). Developing these abilities enables the transformation of individuals into lifelong learners who are better adapted to the changing market conditions and demands of the 21^st^ century. These abilities are crucial for learners and employees in many fields, including the fields of health management and medicine.

This study adds to the existing literature by emphasizing the importance of incorporating digital tools in education, particularly the use of the Padlet platform to facilitate collaborative learning and improve the quality of teaching and learning. It demonstrates that Padlet-mediated online collaborative learning (OCL) can be an effective digital learning tool because of its ease of use and ability to accommodate both pedagogical and technological challenges. This method can be conducted openly or anonymously, depending on the instructor’s preference, and is suitable for students who may be hesitant to participate or fear criticism. Additionally, the accessibility and availability of Padlet make it suitable for use in large classes. It is worth noting that despite the benefits of Padlet-mediated OCL, this methodology may also pose challenges that require careful management and support. This may include gradual integration into the curriculum and ongoing communication between students and the instructor.

## Data availability statement

The raw data supporting the conclusions of this article will be made available by the authors, without undue reservation.

## Ethics statement

The studies involving human participants were reviewed and approved by Hadassah Academic College, Jerusalem, Israel. The patients/participants provided their written informed consent to participate in this study.

## Author contributions

LN-S and DA: conceptualization, data curation, methodology, writing – original draft preparation, and writing – reviewing and editing. All authors contributed to the article and approved the submitted version.

## Conflict of interest

The authors declare that the research was conducted in the absence of any commercial or financial relationships that could be construed as a potential conflict of interest.

## Publisher’s note

All claims expressed in this article are solely those of the authors and do not necessarily represent those of their affiliated organizations, or those of the publisher, the editors and the reviewers. Any product that may be evaluated in this article, or claim that may be made by its manufacturer, is not guaranteed or endorsed by the publisher.
